# A Large Lipoma of the Labia Majora

**DOI:** 10.7759/cureus.20066

**Published:** 2021-11-30

**Authors:** Leena H Moshref, Haifaa Malaekah

**Affiliations:** 1 Department of General Surgery, Doctor Soliman Fakeeh Hospital, Jeddah, SAU; 2 Department of General Surgery, Fakeeh College of Medical Sciences, Jeddah, SAU

**Keywords:** imaging, case report, outcome, labia, lipoma

## Abstract

A 40-year-old woman presented with a huge right labial swelling that had been enlarging gradually for the last four years. A 10-cm labial swelling was revealed on clinical examination. Preoperative magnetic resonance imaging (MRI) was used to rule out other possible diagnoses, such as hernias, vulvar cysts and tumours. When it came to assessing and diagnosing soft tissue swelling, MRI was the method of choice. It determined that the swelling had a bright T1 and T2 signal intensity similar to that of subcutaneous fat, with a decline in signal in fat-saturated sequences, indicating a diagnosis of vulvar lipoma. The mass was surgically excised, and histopathology confirmed the diagnosis of a benign lipoma. Lipomas are considered the most common soft tissue tumours accounting for a prevalence of 1% in the population. Their occurrence in the vulva in premenopausal women is considered rare and only a few cases of vulvar lipomas have been reported in the literature. Herein, we report a rare case of a patient with vulvar lipoma and review the available literature.

## Introduction

Lipomas are considered the most common soft tissue tumours with a prevalence of 1% in the population and an incidence of 2.1/1000 individuals/year [1]. They are benign mesenchymal neoplasms mostly found in the shoulders, nape, upper back, abdomen, buttocks, and proximal portions of the limbs [1]. They commonly emerge between the ages of 40 and 60 [2]. Their occurrence in the vulva in premenopausal women is considered rare, and only a few cases of vulvar lipoma have been recorded in the literature [2-9]. Various pathologies can present as a swelling in the groin, and the definitive diagnosis of inguinal and scrotal lesions could be confusing for numerous physicians due to their comparable clinical presentations [10]. Groin lesions can be classified as neoplastic or non-neoplastic [11]. Neoplastic lesions include lipomas, epidermoid cysts, angiomyofibroblastoma-like tumours, liposarcomas, synovial sarcomas, lymphomas, and metastases from various malignancies/organs, neuroendocrine carcinomas, as well as cancers of the lung, breast, urinary bladder, ovary, vulva, and colon, are examples. Non-cancerous lesions include round ligament varices, hernias, Kimura disease, endometriosis, Castleman disease, inflammation, and hematoma.

Ultrasonography (USG) is thought to be the first imaging modality that should be used for the diagnosis of groin masses [3,4,11]. USG can distinguish between solid and cystic lesions. It can differentiate between the lesions based on their echogenicity, ranging from hyperechoic to hypoechoic, when compared with the surrounding soft tissues. However, it is nearly impossible to differentiate lipoma from liposarcoma by USG alone. Moreover, USG is operator-dependent. Thus, another imaging modality is needed for the preliminary diagnosis of the lesion. Computed tomography (CT) can be helpful in diagnosing lipomas and ruling out other pelvic pathologies as reported by Khreisat et al [7]. Magnetic resonance imagining (MRI) can be helpful if CT findings are inconclusive as it is specific for soft tissues and some case reports name MRI as the imaging modality of choice [3,8]. One case reported using fine-needle aspiration as a preoperative investigation in diagnosing lipoma after initial USG [9]. One study reported not performing preoperative imaging because of the limited resources in their developing country [4]. 

Herein, we report a case of a lipoma of the labia majora that was carefully investigated and successfully excised surgically in a tertiary-care hospital.

The case report has been conducted in line with the Surgical CAse REport (SCARE) criteria [12]. The patient had given her permission for the case and figures to be published.

## Case presentation

A 43-year-old, newly married patient was referred to the general surgery ward from the gynaecology department. She presented with a painless, slowly growing swelling in the right labium for the past four years with no other symptoms. The mass was found as part of the clinical exam done by the gynaecologist as the patient was undergoing investigation for infertility. The patient had believed that this mass was the cause of her infertility. No previous history of trauma was present. She had undergone laparoscopic cholecystectomy in the past.

Examination revealed a single soft 5 x 9 cm mass in the right labium majus, non-tender, non-fluctuant, and irreducible with no transillumination, a negative cough impulse test, and no inguinal lymph nodes enlargement. The overlying skin was normal.

Investigations

**Figure 1:**
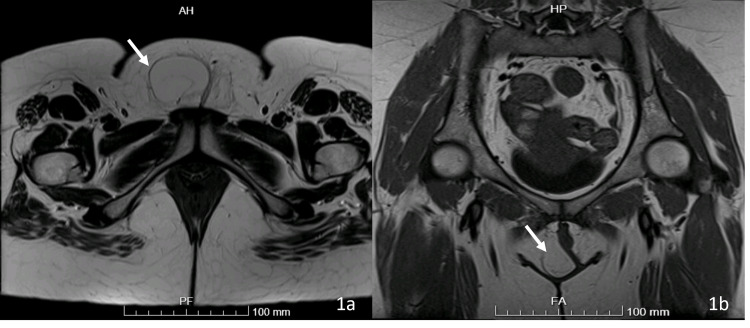
Axial (a) and coronal sections (b) of the magnetic resonance imaging of the pelvic floor.

MRI pelvis (Figure 1) showed a well-defined encapsulated fatty mass centred within the right labium majus with maximum diameters of 4.6 x 4.3 x 10.3 cm in the anterior-posterior, transverse, and craniocaudal directions, respectively. The mass demonstrated bright T1 and T2 signal intensity similar to that of the subcutaneous fat with a drop in signal in the fat-saturated sequences in keeping with a vulvar lipoma. No enlarged inguinal lymph nodes or vulvar cysts were observed. Left labium majus was unremarkable.

Differential diagnosis

Clinically, vulvar lipomas must be differentiated from other lesions such as the cystic enlargement of the canal of Nuck and Bartholin's gland [1,13] and they may be misdiagnosed as inguinal or femoral hernias [10,14]. Vulvar lipomas, like other lipomas, normally tend to have a benign path. If it was not treated, size may increase remarkably [1].

Treatment

**Figure 2:**
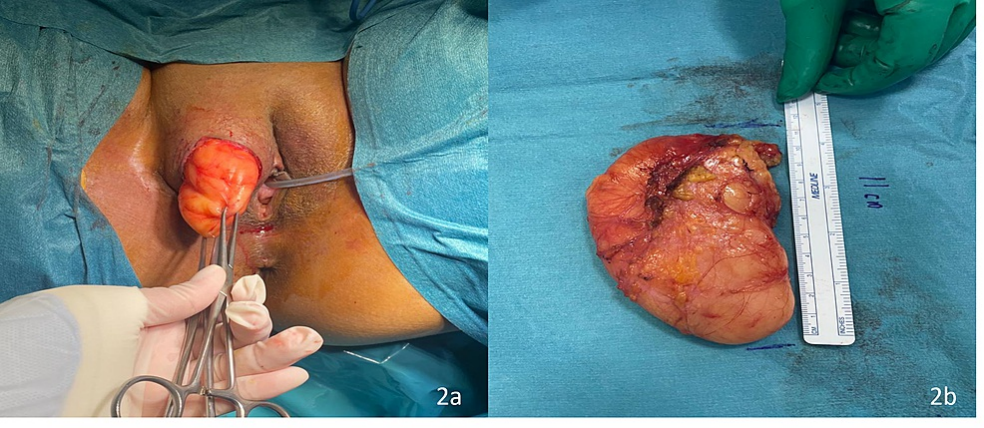
Elliptical incision (a) and gross appearance of lipoma (b).

Surgical excision was planned. The operation was performed under general anaesthesia and the patient was placed in a lithotomy position. Blunt and sharp dissection was carried out, and a mass measuring 11 cm was excised (Figure 2). The vulvar skin was reconstructed and closed in two layers using vicryl 3-0 sutures.

Outcome and follow-up

The patient was discharged on the first postoperative day. Histopathological examination confirmed the diagnosis of a benign lipoma. She was followed up in the clinic after one and four weeks postoperatively. The postoperative course was uneventful, there was no discharge from the surgical site, and sutures were removed.

## Discussion

Vulva benign tumors are characterized according to whether they originate from epithelial or mesenchymal cells [13]. Vulvar lipomas are rare benign mesenchymal tumors made up of mature fat cells with strands of fibrous connective tissue dispersed throughout [12].

Lipomas are most frequent in people in their fourth to sixth decades of life, but they can occur at any age. A vulvar lipoma has a presentation similar to that of other lipomas, reported in any age group [3-5,13].

The aetiology and pathogenesis of lipomas remain unclear. Trauma, obesity, and genetic abnormalities are some of the reported risk factors involved in the development of lipomas [1]. Our patient was in the fourth decade of life and had no other risk factors. Similarly, earlier vulvar lipoma case reports did not include any risk factors [3,4,7,10,15]. Only one report by Lee et al. suggested that lipoma in their adolescent patient might have been linked to trauma [13].

Lipomas typically present as single or multiple soft tissue swellings that gradually growing, non-painful, mobile, non-adherent to the overlying skin with a characteristic doughy feel [1,13]. This is similar to the clinical findings in our case. Therefore, they can be diagnosed correctly in some cases on the basis of clinical examination alone without imaging modalities [4,7].

When the clinical diagnosis is not clear, imaging like USG, CT, and MRI are helpful in distinguishing vulvar lipomas from vulvar cysts, liposarcomas, and inguinal hernias [8,10,13,15]. Vulvar lipomas are nonspecific homogeneous echogenic structures with lobular features that imply fat accumulation on USG [15].

It has been reported that USG is highly sensitive, specific, and reliable [16], and it is the investigation of choice in developing countries as it is less expensive. However, it is operator-dependent. A previous research of USG imaging of superficial lipomas revealed that the majority of patients had homogeneous echotexture and well-defined masses [8]. Lipomas usually have a high signal on weighted pictures, and MRI is the most effective imaging modality for evaluating soft tissue swellings [8].

In determining the anatomical extent of vulvar lipomas and distinguishing them from liposarcomas, CT scans and MRI are useful [13,15]. Nonetheless, as documented in a research by Odoi et al [4], cost and availability restrict their usage in most underdeveloped nations. In our study, an MRI was done to confirm the diagnosis and rule out other differentials, for example, liposarcoma and inguinal hernia.

Histologically, a lipoma must be differentiated from a well-differentiated lipoma-like liposarcoma by substantial tumour dissection [15]. Surgical removal, liposuction, laser therapy, and pharmacological injections are all considered therapeutic options for lipomas in general [3]. Complete surgical excision with the removal of capsules to prevent recurrence remains the treatment of choice for vulvar lipomas [4-9,13,14].

Because the lump was big and situated in an unusual location, we opted for surgical removal to confirm the diagnosis of a lipoma, as the vulva is considered an uncommon location for the formation of this mass.

Lipoma in the vulva is a rare occurrence. A comprehensive imaging study is necessary to rule out alternative diagnosis. The surgical treatment of vulvar lipoma is similar to that of other lipomas. It is unclear if all individuals require excision or whether radiological follow-up is necessary. Furthermore, there is no evidence of long-term follow-up in the literature.

## Conclusions

This study concludes that a labial lipoma is rare and needs to be investigated carefully to exclude other malignant masses like liposarcomas. Since it is painless and slowly growing, patients can have the mass for a long time without needing to seek medical advice. There is a chance that the lesion may harbour a malignancy if it is not investigated and managed appropriately. MRI is considered the imaging modality of choice. MRI is helpful in differentiating benign from malignant soft tissue lesion, hence wide excision is not recommended in cases of benign-looking lipoma. We recommend providing awareness to the women and encouraging them to seek medical advice if they have a vulvar mass.
